# Antibody therapy can enhance AngiotensinII-induced myocardial fibrosis

**DOI:** 10.1186/1755-1536-7-6

**Published:** 2014-04-10

**Authors:** Nicole L Rosin, Alison J Gareau, Devin Betsch, Alec Falkenham, Mryanda J Sopel, Timothy DG Lee, Jean-Francois Légaré

**Affiliations:** 1 Department of Pathology, Dalhousie University, Halifax, Nova Scotia, Canada; 2 Department of Surgery, Dalhousie University, Halifax, Nova Scotia, Canada; 3 Department of Microbiology and Immunology, Dalhousie University, Halifax, Nova Scotia, Canada; 4 Department of Surgery, New Halifax Infirmary, 1796 Summer St., Room 2269, Halifax, NS B3H 3A7, Canada

**Keywords:** Myocardial fibrosis, Collagen, Antibody, Inflammation, AngiotensinII

## Abstract

**Background:**

Myocardial fibrosis is a pathological process that is characterized by disrupted regulation of extracellular matrix proteins resulting in permanent scarring of the heart tissue and eventual diastolic heart failure. Pro-fibrotic molecules including transforming growth factor-β and connective tissue growth factor are expressed early in the AngiotensinII (AngII)-induced and other models of myocardial fibrosis. As such, antibody-based therapies against these and other targets are currently under development.

**Results:**

In the present study, C57Bl/6 mice were subcutaneously implanted with a mini-osmotic pump containing either AngII (2.0 μg/kg/min) or saline control for 3 days in combination with mIgG (1 mg/kg/d) injected through the tail vein. Fibrosis was assessed after picosirius red staining of myocardial cross-sections and was significantly increased after AngII exposure compared to saline control (11.37 ± 1.41%, 4.94 ± 1.15%; *P* <0.05). Non-specific mIgG treatment (1 mg/kg/d) significantly increased the amount of fibrosis (26.34 ± 3.03%; *P* <0.01). However, when AngII exposed animals were treated with a Fab fragment of the mIgG or mIgM, this exacerbation of fibrosis was no longer observed (14.49 ± 2.23%; not significantly different from AngII alone).

**Conclusions:**

These data suggest that myocardial fibrosis was increased by the addition of exogenous non-specific antibodies in an Fc-mediated manner. These findings could have substantial impact on the future experimental design of antibody-based therapeutics.

## Background

Despite advances in medical therapy, cardiovascular disease remains the cause of 33% of all deaths in the United States and 28% in Canada [1]. Furthermore, the 5-year mortality rate for patients presenting with heart failure is 50% [2]. The pathological characteristics and the final pathway leading to heart failure is the development of myocardial fibrosis [3]. Myocardial fibrosis is defined as excess deposition of extracellular matrix (ECM) proteins within the myocardium and is a significant contributor to myocardial dysfunction.

A common animal model of myocardial fibrosis is induction by exposure to AngiotensinII (AngII), a vasoregulatory hormone, which results in hypertension-mediated myocardial fibrosis [4,5]. We, and others, have previously reported that myocardial fibrosis after exposure to AngII is characterized as early as 3 days by myocardial infiltration of mononuclear cells that have been defined as bone marrow-derived, monocyte lineage, fibroblast progenitor cells (fibrocytes) [6-8]. This is followed by a significant increase in extracellular matrix deposition, the hallmark of fibrosis.

Using this model, investigators have identified the pro-fibrotic mediator transforming growth factor-β (TGFβ) as being central to the regulation of fibrosis development. In the AngII-induced model, the TGFβ pathway, including SMAD phosphorylation and CTGF production, are significantly upregulated within the first 3 days, suggesting their involvement in the initial phase of myocardial fibrosis development [4]. In support of the role for TGFβ, inhibition of TGFβ using antibodies [9] or mice heterozygous for TGFβ knockout [10], have resulted in reduced myocardial fibrosis. However, TGFβ is a pleiotropic cytokine that has both pro-inflammatory and anti-inflammatory functions, making treatment strategies based on its reduction difficult, as serious deleterious effects have been reported, such as increased mortality after myocardial infarction [11]. Other antibody-based therapeutic strategies attempted in animal models have included targeting IL23R, platelet-derived growth factor-receptor, and the TGFβ downstream mediator connective tissue growth factor (CTGF), without any antibody-based therapy in clinical use to date [12,13].

In the present study, we show that non-specific antibody, similar to those used as controls for therapies used to treat myocardial fibrosis can in fact worsen the onset of fibrosis in the AngII-induced model. We provide evidence that the mechanism for this apparent paradoxical observation is mediated by increased pro-inflammatory cytokine production and is not dependent on the specificity of the antibody. In fact, the worsened fibrotic response to whole antibody disappeared with treatment using Fab fragments, suggesting that an Fc-mediated activation of infiltrated cells was key to the exacerbated fibrotic response.

## Results

### Effect of antibody on early myocardial fibrosis

**Figure 1:**
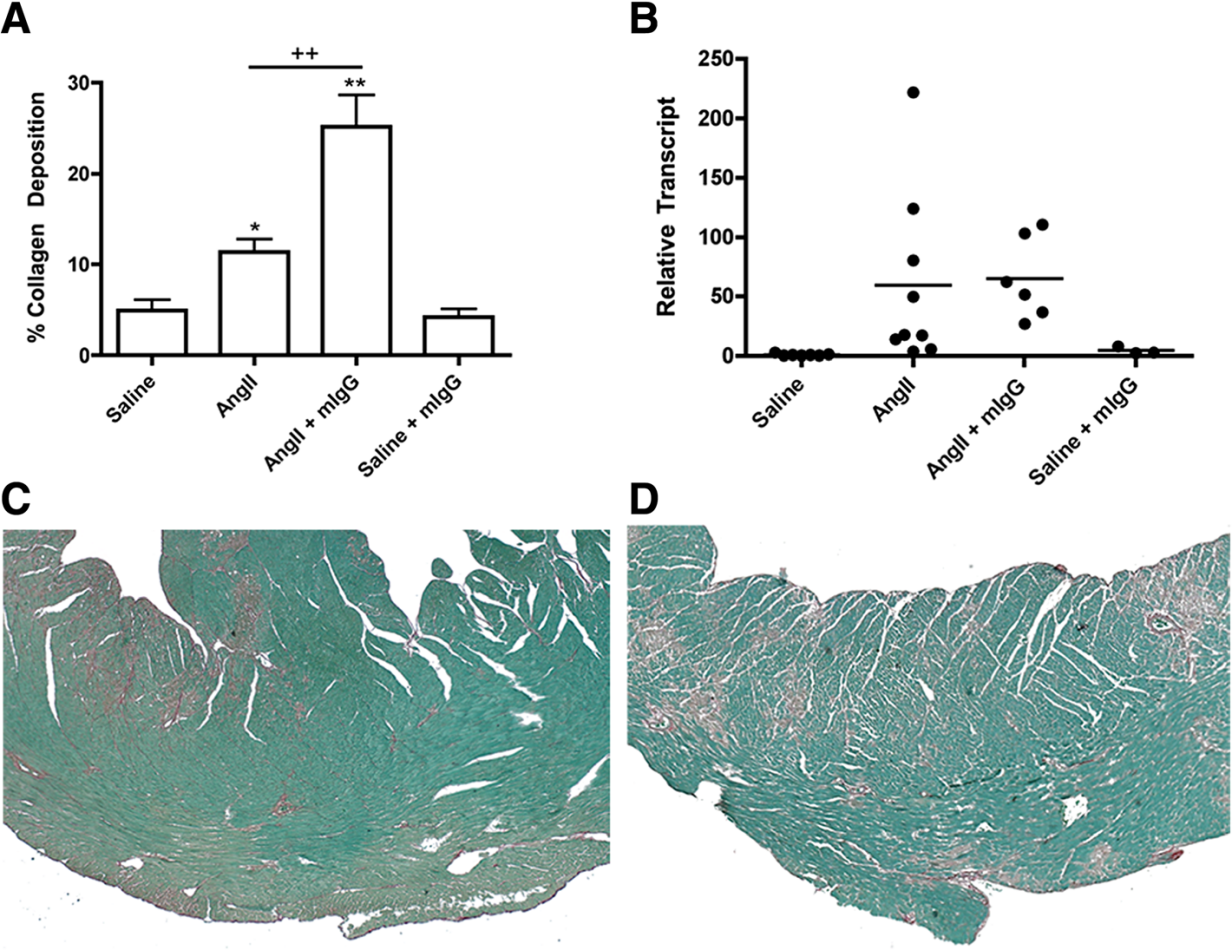
**Antibody administration and fibrosis.** Animals exposed to AngII for 3 days were concurrently administered non-specific mIgG by tail vein injection. Myocardial cross-sections were stained with Sirius red to assess the AngII development of fibrosis. The percentage of collagen deposited within whole cross-sections was assessed using image analysis software **(A)** and qPCR was used to assess myocardial expression of *Col1A1***(B)**. Transcript levels are reported relative to the housekeeping gene 18S. Representative images of AngII **(C)**, and AngII + mIgG **(D)** are shown at 10x. *P <0.05, **P <0.01 compared to saline, ^+^*P* <0.05, ^++^*P* <0.01 compared to AngII.

The addition of non-specific antibody by tail-vein injection to the AngII model of myocardial fibrosis over 3 days resulted in a significant increase of the fibrotic area within the myocardium. The amount of fibrosis was significantly increased after mIgG treatment, with 26.34 ± 3.03% of myocardium affected compared to 11.29 ± 1.20% with AngII alone, and with a more dispersed pattern of collagen deposition (Figure 1A, C, D). The mRNA expression of *Col1A1*, a subunit of type I collagen, the most prolific structural collagen in the myocardium, also increased in the myocardium of animals exposed to AngII, but did not increase further in the AngII + mIgG group (Figure 1B).

**Figure 2:**
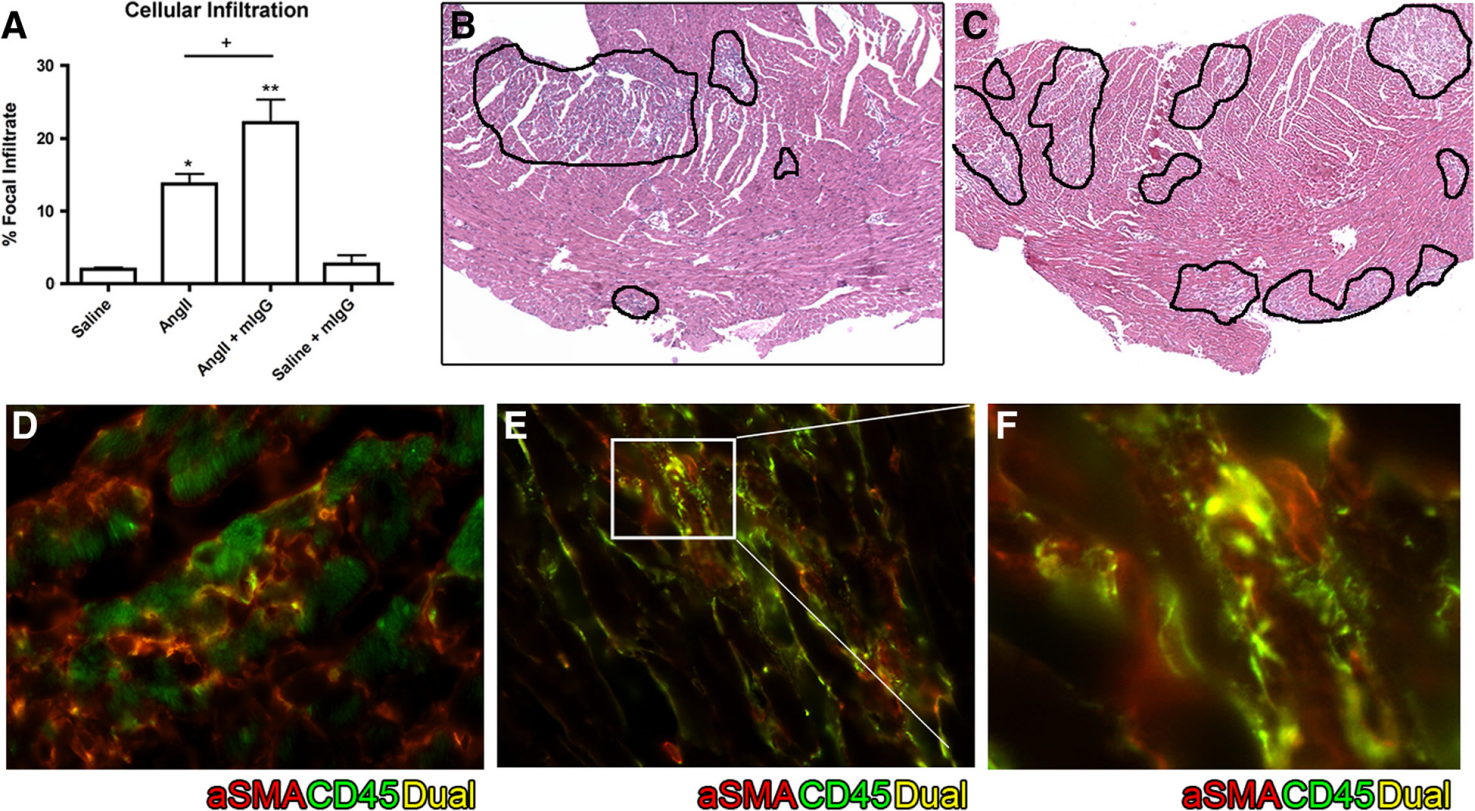
**Antibody administration and cellular infiltration.** Animals exposed to AngII for 3 days were concurrently administered mIgG by tail vein injection. The amount of cellular infiltrate within whole cross-sections was assessed using a grid-scoring method of whole myocardial cross-sections after H&E stain and the results reported as percentage of area affected **(A)**. Representative H&E stained sections of AngII **(B)** and AngII + mIgG **(C)** are shown at 5x and areas of infiltration are highlighted by black borders. Immunofluorescent staining of CD45 (green) and SMA (red) indicate dual staining (yellow) within areas of infiltration in AngII **(D)**, AngII + mIgG **(E)** treated myocardium, with representative images shown at 63x and an additional 4x digital magnification of an area of dual staining shown **(F)**. *P <0.05, **P <0.01 compared to saline, ^+^*P* <0.05, ^++^*P* <0.01 compared to AngII.

Similarly, the amount of cellular infiltration assessed using a standardized grid-affected method after staining whole myocardial cross-sections with hematoxylin and eosin (H&E) was significantly higher in animals receiving the non-specific mIgG (24.42 ± 3.67%) when compared to AngII alone (14.27 ± 1.22; *P* <0.01) (Figure 2A, B, C). The phenotype of the cellular infiltrate in both groups included cells expressing both α-smooth muscle actin (SMA) and CD45 in keeping with a phenotype for fibrocytes that co-express monocyte and mesenchymal markers [14]. Fibrocytes, have been suggested to be precursors to myofibroblasts and as such important ECM producing effector cell seen in fibrotic disorders ([15]) (Figure 2D, E, F). Taken together, our findings demonstrate that antibody addition to AngII-exposed animals in fact results in worsening the fibrotic changes within the myocardium.

### Increased fibrosis is not due to increased complement activation

**Figure 3:**
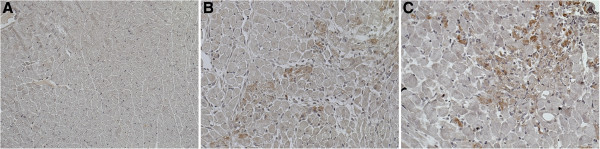
**Complement activation.** Immunohistochemical staining against C4d was used to assess the amount of complement activation in myocardium of animals exposed to saline **(A)**, AngII **(B)**, and AngII + mIgG **(C)** for 3 days. Representative images are shown at 20X.

A potential mechanism by which the addition of mIgG exacerbated fibrosis is complement-mediated cytotoxicity. In order to assess if complement is involved we stained cross-sections of myocardium using anti-C4d antibody. In the AngII alone and AngII + mIgG groups we observed positive C4d staining, primarily located in areas of cellular accumulation within the myocardium (Figure 3A, B, C). Given the similar amount of C4d in both AngII and AngII + mIgG groups, this suggests that complement activation was not specific to the addition of mIgG. Further support for the lack of deposition of IgG in the myocardium is provided by a lack of identifiable immunohistochemical staining for IgG within the myocardium of any of the groups (Additional file 1). Taken together, our findings indicate that the increased fibrotic response with the addition of mIgG is not due to complement activation.

### Increased fibrosis is secondary to increased pro-inflammatory cytokines within the myocardium

**Figure 4:**
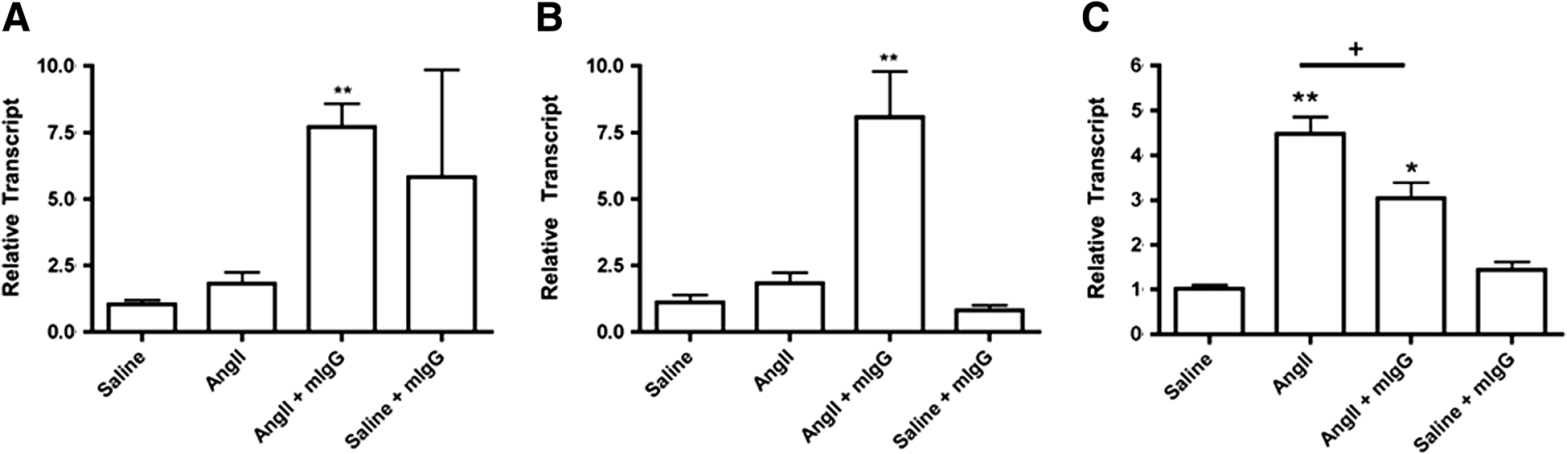
**Pro-inflammatory regulation.** qPCR was used to assess myocardial expression of *TNFα***(A)**, *IL1β***(B)**, and *TGFβ***(C)** after saline, AngII, or AngII + mIgG exposure for 3 days. Transcript levels are reported relative to the housekeeping gene *18S*. *P <0.05, **P <0.01 compared to saline, ^+^*P* <0.05, ^++^*P* <0.01 compared to AngII.

The overall increase in mononuclear cells, many of which expressed markers of fibrocytes (CD45^+^/SMA^+^) within the myocardium in the AngII + mIgG group suggests that inflammatory signaling may be contributing to the enhanced cellular response to AngII. We therefore assessed the mRNA expression of two known potent inflammatory mediators, tumour necrosis factor-α (TNFα) and interleukin 1β (IL1β) within the myocardium [16,17]. *TNFα* and *IL1β* mRNA expression were not significantly affected by exposure to AngII alone compared to saline, but increased significantly with the addition of mIgG (TNFα 7.72 ± 0.87 fold; IL1β 8.08 ± 1.72 fold; *P* <0.01) (Figure 4A, B). We then looked at TGFβ as a predominant pro-fibrotic cytokine normally up-regulated in animals exposed to AngII [18,19]. As previously shown, there was a significant increase in *TGFβ* mRNA expression in the myocardium of AngII animals relative to saline control (Figure 4C). However, in animals that received AngII + mIgG there was a significant reduction in *TGFβ* expression when compared to AngII alone that remained higher than in saline control (3.05 ± 0.35 *vs.* 4.48 ± 0.38 fold) (Figure 4C). Our findings suggest that the addition of mIgG may be favoring pro-inflammatory signaling (TNFα and IL1β) as opposed to anti-inflammatory signaling (TGFβ), contributing to the activation of the cells within the myocardium and ultimately resulting in increased fibrosis.

### Fibrocytes enriched from PBMCs are activated by mIgG

**Figure 5:**
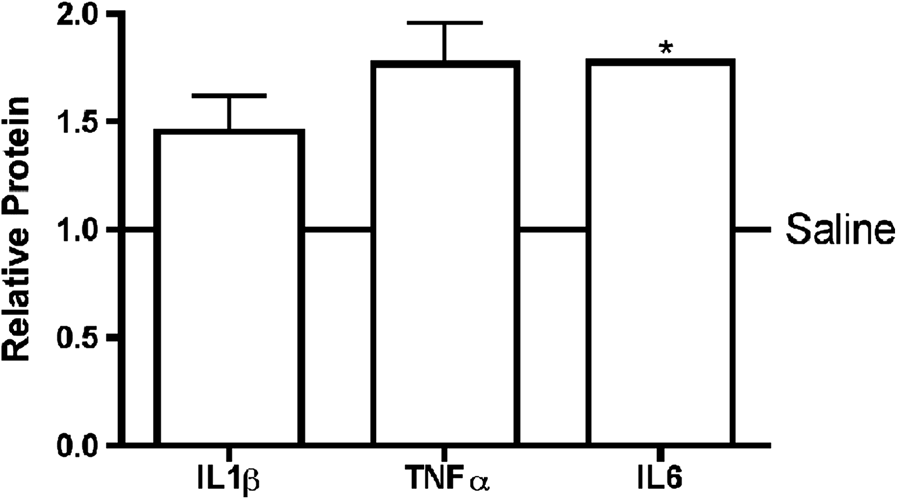
**PBMC stimulation with mIgG.** Isolated PBMCs were exposed to mIgG at 25 mg/mL. Supernatants were collected and ELISAs were used to assess for TNFα, IL1β, and IL6 production, reported relative to saline control (solid line). *P <0.05 compared to saline.

We have previously demonstrated that a large portion of infiltrating cells after AngII exposure are monocyte-derived fibrocytes and that isolated circulating peripheral blood mononuclear cells (PBMCs) can be made to differentiate into fibrocytes under specific conditions [4,20]. This explains in part why we used human PBMCs in addition to their easy availability to evaluate the impact of exposure to IgG. One should note that human and murine FcγR subclasses are not homologous, but are able to bind IgG that originate from the opposite species as previously shown by others, which supports our experimental approach [21,22]. We therefore incubated PBMCs *in vitro* with mIgG for 48 h. This exposure resulted in increased production of the pro-inflammatory cytokines IL1β, TNFα, and IL6 measured by ELISA with IL6 reaching significance compared to saline control (Figure 5). This suggests that mononuclear infiltrating cells originating from the circulation can be directly affected by the presence of mIgG, suggesting a potential mechanism for our observed increase in fibrosis *in vivo*. In support of our findings is previously published evidence that IgG can activate mononuclear cells, resulting in the differentiation of monocytes and the production of pro-inflammatory cytokines [23,24].

### Increased fibrosis seen in animals receiving mIgG is prevented by using Fab fragments or mIgM

**Figure 6:**
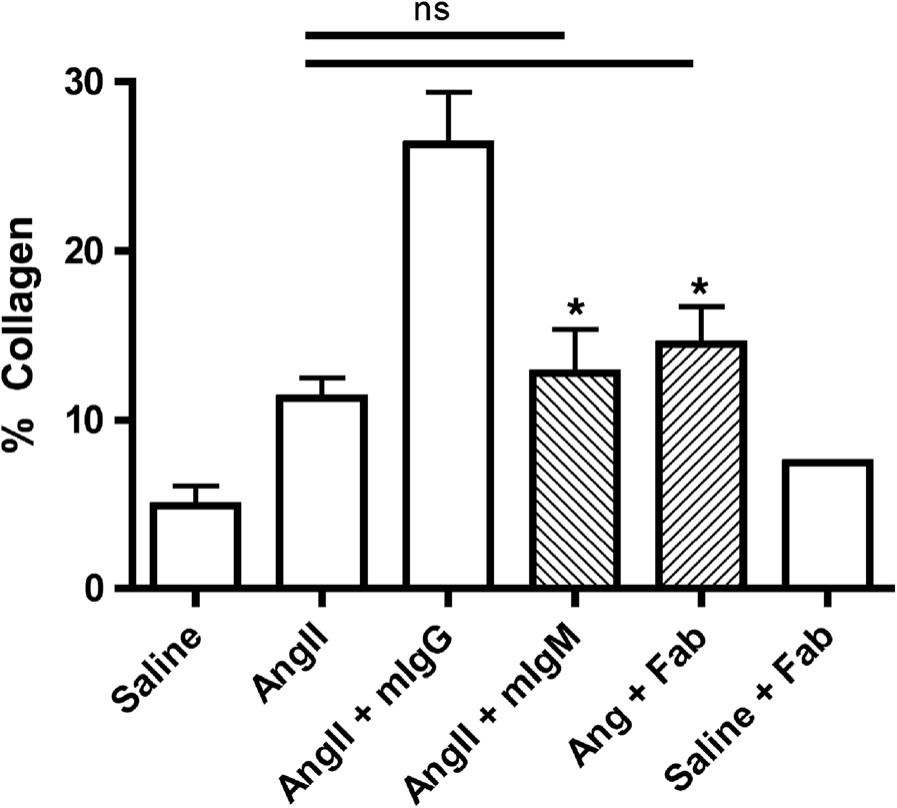
**AngII plus Fab or IgM.** Animals were exposed to AngII for 3 days and concurrently Fab fragments or IgM were injected by tail vein for a total of 3 days. Fibrosis was semi-quantified by image analysis software of Sirius red stained whole cross-sections of myocardium. *P <0.05 compared to saline.

Fc receptor recognition and binding of the Fc fragment of IgG molecules leads to increased inflammatory signaling [25]. We therefore removed the Fc portion from the mIgG molecules, and treated AngII exposed animals with the resulting Fab fragment (Figure 6). When AngII exposed animals were treated with Fab, myocardial fibrosis was significantly reduced compared to AngII exposed animals treated with whole mIgG (14.49 ± 2.23 *vs.* 26.34 ± 3.03%; *P* <0.05). In order to further support our observation we tested the impact of adding whole mIgM to AngII exposure. IgM is not recognized by FcγRs, and so does not activate mononuclear cells in the circulation in an Fc-mediated manner [25]. Similar to animals that received AngII + Fab, mice exposed to AngII + mIgM did not have elevated fibrosis compared to animals exposed only to AngII. Importantly, fibrosis was significantly reduced compared to the AngII + mIgG group (12.74 ± 2.59%). Taken together, these findings suggest that the mechanism behind the exacerbation of fibrosis was Fc-mediated.

## Discussion

There are no existing therapies available to treat established myocardial fibrosis. In the present study, animals exposed to AngII were administered a non-specific mouse IgG, such as would be used as a control in targeted antibody therapies for fibrotic diseases. Surprisingly, the introduction of antibody to this model of myocardial fibrosis increased the amount of fibrosis in the first few days of exposure. Our observations were robust as we provided direct correlation of increased fibrosis with increased cellular infiltration, as previously described [6]. We also used two different preparations of mIgG antibodies with similar results further supporting the observations (reported results were pooled). We therefore sought to investigate the potential mechanisms by which mIgG given to animals could affect myocardial fibrosis. This is particularly relevant as investigators are currently working on a multitude of targets using antibody approaches.

Evidence for the direct binding of the IgG molecules within the myocardium was assessed, as this could have contributed to antibody-dependent complement mediated cytotoxicity [26]. Complement activation occurs when complement component 1 recognizes surface bound IgG, leading to initiation of a cascade that ultimately results in cell lysis and deposition of the split product C4d [27]. C4d deposition is a well-described histological technique to identify areas of classical complement activation [26]. We found evidence of complement activation based on C4d deposition in all animals exposed to AngII with no evidence of significant differences between groups in which antibody was added. This suggests that while complement may be involved in AngII-induced myocardial fibrosis, it was not responsible for the increase in fibrosis observed after the addition of antibody. We did not explore the lectin pathway of activation in which complement activation occurs without immunoglobulin.

The AngII exposure model of myocardial fibrosis is one in which myocardial inflammation potentially plays a large role based on early myocardial inflammatory cell migration and pro-inflammatory effects of AngII [6,28]. Particularly relevant are monocyte-derived fibrocytes that have been identified as an important cell type in the development of AngII-mediated myocardial fibrosis [6,8]. We observed that in animals exposed to AngII and antibody there was a significant increase in the pro-inflammatory cytokines TNFα and IL1β. This is particularly relevant as these cytokines are produced primarily by monocytes and macrophages, which can be activated by the binding of IgG to Fc gamma receptors I and IIA (FcγRs) [25,29,30]. Mouse (and human) FcγRs have higher affinity for certain IgG subclasses, which have been extensively reviewed by others [21]. The administration of whole IgG containing all subclasses in the present study implies that we do not know which FcγR is recognizing the exogenous IgG, or if it is an activating or inhibitory receptor subtype that is involved. However, as both macrophages and fibrocytes are monocyte-derived cells, it follows that mIgG may also be mediating the activation of fibrocytes that infiltrate the myocardium in our model. To test this hypothesis, we exposed PBMCs containing monocytes to mIgG. Using this *in vitro* assay we were able to show that the addition of mIgG can activate PBMCs influencing the production of pro-inflammatory cytokines IL1β, TNFα, and IL6.

Another potential mechanism of action of mIgG that could enhance the AngII-induced inflammatory response is through the ability of IgG to form aggregates [31]. The FcγRs preferentially bind monomeric (FcγI) or aggregate IgG (FcγRII, III) [32]. In general, protein aggregates interact with B cell receptors, activating B cells or targeting protein to major histocompatibility complex type II [33]. Additionally, IgG aggregates can cause clustering of FcγRs, contributing to inflammation [31]. The presence of only a small fraction of aggregated protein is capable of eliciting a response [33]. In the current study risk factors for aggregation were avoided; preparations were not heated or stored in conditions reported to contribute to protein aggregate formation and preparations were filtered prior to injection [33]. In addition, the mIgG was stored at 2 mg/mL, and the dosage utilized for IV administration was 0.25 mg/mL, both of which are well below the concentration at which IgG spontaneously forms aggregates (30 mg/mL) [31]. Further support for the response elicited after mIgG administration in the current study is provided by the reported inhibition of fibrocyte differentiation by aggregated IgG [32].

The treatment of AngII-exposed mice with only the Fab fragment of the mIgG, which were produced by removing the Fc portion and therefore the antibodies ability to signal through FcγRs did not result in an increase in fibrosis development compared to AngII alone. Similar results were observed when animals were exposed to IgM in addition to AngII, supporting our hypothesis that the observed increase of fibrosis with IgG was based on the Fc portion interactions. Taken together, we have shown that the use of mIgG in animals receiving AngII can enhance the primary inflammatory reaction within the myocardium and this appears to be mediated by Fc-mediated activation of monocyte-derived cells.

One should note that the present study was not designed to characterize the exact phenotype of infiltrated cells or whether fibrocytes or macrophages were responsible for Fc activation. However, Pilling et al. previously reported that the blockage of FcγR reduced the ability of circulating PBMCs to differentiate into fibrocytes, linking Fc activation to infiltrated cells in models of myocardial fibrosis [20]. It is also possible that stimulation of FcγRs may have a role in increasing macrophage differentiation from a pro-inflammatory to anti-inflammatory/pro-fibrotic state, ultimately resulting in an increase in fibrosis [29]. However, this has yet to be confirmed in the AngII model of myocardial fibrosis. Furthermore, our findings suggest that mIgG in combination with AngII was associated with less TGFβ, suggesting a reduction in the anti-inflammatory/pro-fibrotic phenotype. This would result in an important change in the cytokine environment and balance between inflammation/cellular infiltration and resolution, potentially resulting in increased fibrosis.

While there is no established anti-fibrotic therapy in clinical use, there are a number of previous studies that have illustrated the potential applicability of neutralizing antibodies to reduce fibrosis in animal models. Such studies often report only changes between specific, targeted antibody and the non-specific control antibody treatments [34,35]. However, others have reported data suggesting that there are differences between AngII alone and AngII + IgG control antibody treatments, although these differences, such as in number and type of infiltrating cells were not investigated in depth [12].

## Conclusions

The present study raises important considerations in the design of future antibody-based therapies for conditions in which inflammation can have a profound role. Our findings suggest that the Fc portion of the antibody may significantly upregulate inflammation, influencing any therapy used. This could also explain in part, why prior attempts in clinical trials using systemic administration of antibody for fibrotic disorders have largely failed [36-38]. These trials have targeted TGFβ, TGFβRII, TIMP1, and TNFα, which had promising anti-fibrotic results with *in vitro* and *in vivo* animal models [13,39,40].

In summary, we were able to demonstrate that non-specific antibody administered to animals exposed to AngII can result in worsening of myocardial fibrosis by increasing pro-inflammatory cytokine production in the myocardium via Fc portion mediated activation.

## Methods

### Animals

All work was performed in accordance with the Canadian Council on Animal Care and approved by the local Dalhousie’s University Committee on Laboratory Animals. Male C57Bl/6 mice between 7 and 8 weeks of age were purchased from the Jackson Laboratory (Bar Harbour, ME, USA) and were housed within the Carleton Animal Care Facility at Dalhousie University. Mice were provided food and water *ad libitum* for 1 week prior to experimentation.

### Antibody preparation

Two preparations of mIgG were used and the results were pooled as there was no significant difference between the preparations for any of the parameters tested. Non-specific mIgG antibody was purchased from Sigma or isolated from mouse serum. Whole mIgG was fragmented and Fc portion removed using the Pierce Fab Preparation kit (Fisher), following the manufacturer’s instructions. In brief, mIgG in PBS was prepared in dilution buffer and desalted. The mIgG was then mixed with agarose-immobilized papain in a spin column and incubated for 3 h followed by centrifugation to elute digested antibody. The Fab portion was purified from the digest using a Protein A column, after which Fc fragments and remaining whole IgG were eluted. Protein concentrations were measured by absorbance at 280 nm using a Nanodrop 2000 spectrophotometer (Thermo Fisher), and all elution fractions were run on a non-reducing gel to assess purity (Additional file 2).

### AngII infusion and immunoglobulin intravenous injection

Animals were randomly assigned to the following groups; 1- AngII (2.0 μg/kg/min; Sigma Aldrich, Oakville, ON, Canada; n = 7), 2- AngII + mIgG (1 mg/kg/d; n = 6), 3- AngII + Fab (1 mg/kg/d; n = 4), 4- AngII + mIgM (1 mg/kg/d; Biolegend, San Diego, CA, USA; n = 4) and additional saline controls (saline+/-mIgG or Fab; n = 10). Animals had mini osmotic pumps containing AngII or saline implanted under general anesthesia as previously reported [6]. In short, animals were anesthetized with isoflurane (Baxter Healthcare Corp., New Providence, NJ, USA) in oxygen at which point a 1 to 2 cm mid-scapular skin incision was made and a mini osmotic pump (Alzet, Palo Alto, CA, USA) containing AngII or saline was inserted subcutaneously. The pumps remained in for 3 days during which the animals were provided food and water *ad libitum* and observed for signs of morbidity. Mouse IgG, Fab, and IgM were diluted in sterile saline and equalized to 100 μL, which was injected via the tail vein daily starting on the day of pump insertion surgery, for a total of three doses.

### Tissue harvest

Hearts from experimental animals were harvested and weighed, then divided into three sections. The base section of the heart was processed for histological examination with the apical section split vertically and these pieces were snap frozen in liquid nitrogen immediately for molecular analysis.

### Histological analysis

Hearts were processed for histological assay by fixing with 10% formalin for 24 h or protecting with sucrose/OCT followed by snap freezing. Formalin fixed tissue was paraffin-embedded and serially sectioned on a microtome (5 μm). Basic myocardial histology and cellular infiltration were examined using heart cross-sections stained with hematoxylin and eosin. A blinded observer quantified the infiltrating cells by counting the number of grids affected within an image of an entire heart cross-section at 50x magnification (1 section per animal), based on a previously published grid-scoring method to quantify the degree of cellular infiltration between groups [6]. Collagen was detected using Sirius red and fast green stains and was semi-quantified as previously described [4]. Briefly, the entire heart cross-section at 5x was analyzed using image analysis software to calculate the percentage of red pixels over the area of the cross-section.

Immunohistochemistry for C4d (Hycult Biotech, Plymouth Meeting, PA, USA) was performed on paraffin embedded tissues, which were deparaffinized and treated for antigen retrieval prior to staining. Briefly, endogenous peroxidases were quenched with 3% hydrogen peroxide; endogenous biotin was blocked (DAKO Biotin Blocking System, DakoCytomation); and non-specific staining was blocked with normal goat serum. Sections were incubated with primary antibody, followed by a specific biotin-conjugated secondary antibody. The antibody complexes were then conjugated to an Avidin-biotin complex (Vectastain ABC kit; Vector, Burlington, CA, USA) and developed using 3,3′ diaminobenzidine as the chromogen (DAB; DakoCytomation). Light microscopy was performed and pictures were captured using a Zeiss AxioplanII with an Axiocam HRC colour camera. Images were analyzed in Adobe Photoshop 5.0.

### Immunofluorescence staining

Sucrose protected, frozen sections were fixed in 4% paraformaldahyde, permeabilized with 0.03% Triton X-100, blocked against non-specific antibody binding with 10% normal goat serum, and stained for CD45 (BD Biosciences, Mississauga, ON, Canada) and alpha-smooth muscle actin (SMA; Sigma). Antibodies were detected using anti-host specific Alexa fluorescently labeled secondary antibodies (Invitrogen). Images were captured with a Zeiss Axiovert 200 inverted microscope with a Hamamatsu ORCA-R2 digital camera with an AttoArc 2 HBO 100 W lamp.

### Relative quantitative polymerase chain reaction (qPCR)

Total RNA was isolated from snap frozen heart sections using TRIzol (Invitrogen, Carlsbad, CA, USA) according to the manufacturer’s protocol. First strand cDNA was synthesized from RNA using iScript cDNA Synthesis Kit (Biorad, Hercules, CA, USA). The qPCR was completed using iQ SYBR Green Supermix (Biorad) and the iQ Multicolour Real-Time PCR Detection System thermocycler (Biorad) was used for detection. Efficiency curves and no-template control samples were run with each thermocycling. Melt curves were run after cycling to ensure target specificity. Primers were designed against mRNA sequences using PrimerBlast [41] and are listed. *TGFβ*, forward 5′-GTCTCCCAAGGAAAGGTAGG-3′, reverse 5′-CTCTTGAGTCCCTCGCATCC-3′; *CTGF*, forward 5′-TCAACCTCAGACACTGGTTTCG-3′, reverse 5′-TAGAGCAGGTCTGTCTGCAAGC-3′; *Col1A1*, forward 5′-CAACAGTCGCTTCACCTACAGC-3′, reverse 5′-GTGGAGGGAGTTTACACGAAGC-3′; *TNFα*, forward 5′-TCTCATGCACCACCATCAAGGACT-3′, reverse 5′-ACCACTCTCCCTTTGCAGAACTCA-3′; *IL1β*, forward 5′-TCCTCGGCCAAGACAGGTCGCT-3′, reverse 5′-CCCCCACACGTTGACAGCTAGGT-3′; *18S* (control), forward 5′-TCAACTTTCGATGGTAGTCGCCGT-3′, reverse 5′-TCCTTGGATGTGGTAGCCGTTTCT-3′. Expression was normalization to the 18S ribosomal gene using the Pfaffl method.

### Cell isolation and culture

PBMCs were isolated from healthy volunteers. The average donor age was 27.3 ± 2.5 years and the group included four men and eight women, none of who had any history of hypertension, cardiovascular disease, or treatment for hypertension. Briefly, whole blood was collected in EDTA containing Vacutainer tubes, diluted in Dulbecco’s phosphate buffered saline (dPBS; Invitrogen) containing 2% fetal bovine serum (FBS; Invitrogen) and processed over Ficoll-Paque Plus gradient (GE Healthcare, Waukesha, WI, USA). The PBMC containing layer was isolated, washed, and cells plated in RPMI media with 10% FBS, 2 mM L-glutamine, 100 mg/mL streptomycin, and 100 U/mL Penicillin.

Isolated PBMCs were treated with mIgG (25 μg/mL) for 48 h. Supernatant was collected, centrifuged to remove cell debris and then stored at -80°C. Relative production of TNFα, Ilβ, and IL6 were assessed using commercial ELISA kits, following the manufacturer’s instructions (IL1β and TNFα, Abcam; IL6, BD Biosciences, Mississauga, ON, Canada).

### Statistical analysis

Data are represented as mean ± SEM. One-way ANOVA tests were completed using the Bonferroni post-test to compare the experimental groups to the relative controls. All qPCR results were evaluated based on the one-tailed *T*-test to compare changes in relative mean expression. All statistical calculations were computed using GraphPad Prism4 software and significance was determined if *P* ≤0.05.

## Abbreviations

AngII: Angiotensin II; CTGF: Connective tissue growth factor; ECM: Extracellular matrix; H&E: Hematoxylin and eosin; IL1β: Interleukin 1β; IL6: Interleukin 6; mIgG: Mouse immunoglobulin G; mIgM: Mouse immunoglobulin M; PBMC: Peripheral blood mononuclear cell; qPCR: Relative quantitative polymerase chain reaction; SMA: α-smooth muscle actin; TGFβ: Transforming growth factor-β; TNFα: Tumor necrosis factor-α.

## Competing interests

The authors declare that they have no competing interests.

## Authors’ contributions

NR designed the study, acquired and interpreted data, and drafted the manuscript. AG assisted in study design, assisted with data acquisition and interpretation, and assisted in drafting the manuscript. AF and MS participated in data acquisition and analysis. DB assisted in design of *in vitro* experiments and acquired related data. Additionally, critical revision of the manuscript was carried out by MS, AF, and DB. TL assisted in study design and critical review of the manuscript. JFL participated in study design and drafting of the manuscript. All authors have approved the final version of the manuscript.

## Supplementary Material

Additional file 1 **Anti-IgG immunohistochemistry.** Immunohistochemical staining against mIgG was used to assess the amount of bound IgG in myocardium of animals exposed to saline **(A)**, AngII **(B)**, and AngII + mIgG **(C)** for 3 days. Representative images are shown at 20x. Click here for file

Additional file 2 **Non-reducing gel of digested Fab.** Non-reducing PAGE (10%) was run to assess the quality of digestion of whole mIgG and isolation of the Fab fragment. Click here for file
