# Supplementary figures and images for: Antibody therapy can enhance AngiotensinII-induced myocardial fibrosis
Source: Fibrogenesis Tissue Repair. 2014 Apr 10;7:6. doi: 10.1186/1755-1536-7-6 (PMC4021636; doi:10.1186/1755-1536-7-6)

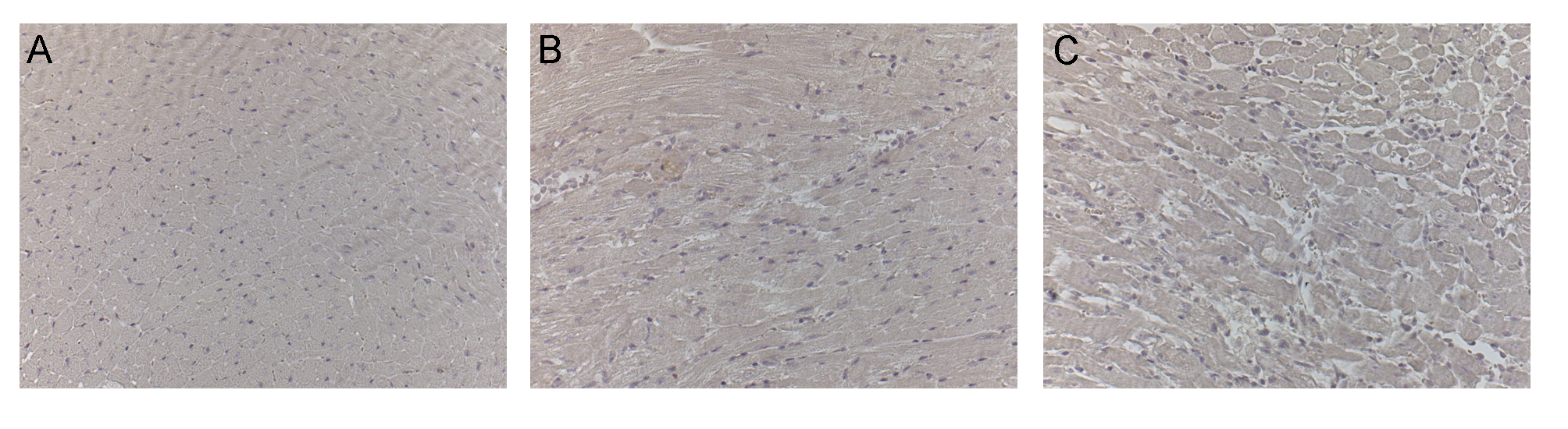

Supplement: Additional file 1 — Anti-IgG immunohistochemistry. Immunohistochemical staining against mIgG was used to assess the amount of bound IgG in myocardium of animals exposed to saline (A), AngII (B), and AngII + mIgG (C) for 3 days. Representative images are shown at 20x. [file 1755-1536-7-6-S1.tiff]

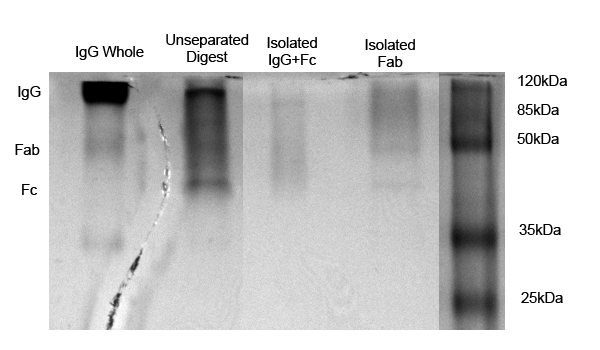

Supplement: Additional file 2 — Non-reducing gel of digested Fab. Non-reducing PAGE (10%) was run to assess the quality of digestion of whole mIgG and isolation of the Fab fragment. [file 1755-1536-7-6-S2.tiff]
